# Coexistence of acute post-streptococcal glomerulonephritis and acute rheumatic fever: case report and systematic review

**DOI:** 10.1007/s00467-026-07189-7

**Published:** 2026-02-17

**Authors:** Tolga Kasap, Ahmet İrdem, Selçuk Yüksel

**Affiliations:** 1 Department of Pediatrics, Çanakkale Onsekiz Mart University, Çanakkale, Türkiye; 2 Faculty of Medicine, Department of Pediatric Cardiology, Çanakkale Onsekiz Mart University, Çanakkale, Türkiye; 3 Faculty of Medicine, Department of Pediatric Rheumatology and Pediatric Nephrology, Çanakkale Onsekiz Mart University, Çanakkale, Türkiye

**Keywords:** Acute rheumatic fever, Acute poststreptococcal glomerulonephritis, Systematic review

## Abstract

**Introduction:**

Acute rheumatic fever (ARF) and acute poststreptococcal glomerulonephritis (APSGN) are serious non-suppurative complications of group A β-hemolytic streptococcal infections. Although they are traditionally linked to distinct “rheumatogenic” and “nephritogenic” strains and have different immunopathogenesis, their simultaneous occurrence in the same patient is extremely rare and the mechanism has not been fully elucidated.

**Case presentation:**

In this study, a 15-year-old male patient who developed ankle pain and edema of the eyelids and extremities following a history of throat infection is presented. Laboratory findings were consistent with nephritic syndrome and elevated ASO levels, and echocardiography revealed rheumatic carditis with multivalve involvement. The patient was diagnosed with concurrent APSGN and ARF, and was treated with benzathine penicillin, oral prednisolone, furosemide, and antihypertensive therapy. While kidney parameters returned to normal during follow-up, mild valvular regurgitation findings persisted.

**Methods:**

Systematic review research was conducted in the following four databases: PubMed, Scopus, Web of Science and Google Scholar published from inception to September 15, 2025. A total of 36 cases were analyzed for clinical and laboratory characteristics, treatment protocols, and follow-up results. The presented case was evaluated comparatively with these data.

**Study eligibility criteria:**

Studies meeting the following criteria were included: (1) full-text articles available in English, (2) case reports, case series or letters to the editor, and (3) information provided on patient demographics, clinical presentation, laboratory findings, treatment and follow-up results. Articles that did not meet these criteria or were review articles without original case data were excluded.

**Results:**

Of the 36 cases analyzed, 86.1% were pediatric. The most common clinical findings were edema (63.8%), fever (55.5%), and hypertension (55.5%). The most common cardiac finding was mitral regurgitation (32/36, 88.8%). Corticosteroids were used in 16/36 cases (44.4%). Kidney recovery was reported in 26/27 cases with follow-up (96.2%), whereas persistent cardiac sequelae were reported in 19/27 cases (70.3%), with mitral regurgitation being the most frequent residual lesion (16/27, 59.2%).

**Conclusion:**

The simultaneous occurrence of APSGN and ARF is a rare but clinically important entity requiring a multidisciplinary approach. While the kidney prognosis is generally favorable, cardiac sequelae frequently persist and may progress. Therefore, after acute nephritic manifestations are controlled, clinical attention should shift to long-term cardiac surveillance and secondary prevention; in patients presenting with APSGN, clinicians should maintain a low threshold for echocardiography when clinical, electrocardiographic, or systemic findings raise suspicion for concurrent rheumatic carditis.

**Graphical Abstract:**

A higher resolution version of the Graphical abstract is available as Supplementary information.

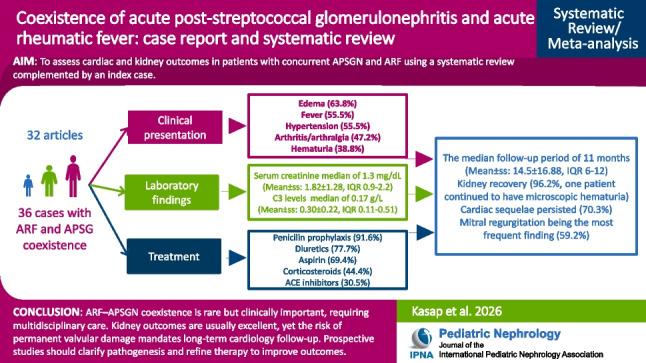

**Supplementary Information:**

The online version contains supplementary material available at 10.1007/s00467-026-07189-7.

## Introduction

Acute rheumatic fever (ARF) and acute poststreptococcal glomerulonephritis (APSGN) are rare but serious non-suppurative complications of group A β-hemolytic streptococcal infections. ARF is usually characterized by findings such as carditis, polyarthritis, Sydenham chorea, subcutaneous nodules, and erythema marginatum, and can cause permanent damage, particularly to the heart valves [1]. APSGN occurs due to immune complex-mediated damage to the kidneys and usually presents with clinical findings such as edema, hypertension, hematuria and proteinuria [2]. Although permanent kidney damage may occur in some patients with APSGN, it is generally known to have a good prognosis, especially in the pediatric age group.

The immunopathogenesis of ARF and APSGN differs; nevertheless, they rarely occur simultaneously in the same patient. The exact mechanism of this condition is not known, but some streptococcal strains may have overlapping rheumatogenic and nephritogenic potential, suggesting that the traditional strain dichotomy may not be absolute [3, 4]. The association of APSGN and ARF has been reported very rarely in the literature, and the clinical diagnosis of this rare condition is usually possible with detailed clinical evaluation and laboratory tests.

The coexistence of APSGN and ARF may increase the risk of serious complications in both cardiac and kidney function. Therefore, it is crucial for clinicians to recognize this rare condition early and implement appropriate treatment strategies. The risk of permanent organ damage can be reduced and long-term prognosis improved, particularly with timely intervention [5, 6].

This article presents in detail the clinical features, diagnostic process, and treatment management of a 15-year-old male patient with the rare concurrent occurrence of APSGN and ARF, alongside a literature review of similar reported cases to provide a comprehensive perspective on this association. In addition, this systematic review updates the published evidence through September 15, 2025 and provides a focused synthesis of clinical presentation, treatment strategies and long-term kidney and cardiac outcomes.

## Case presentation

A 15-year-old male patient presented to the hospital with complaints of swelling in the eyelids, hands, and feet, as well as pain in the ankle. His medical history revealed a sore throat lasting a few days approximately 15 days prior to admission, followed by the onset of swelling in the hands, feet, and eyelids over the past four days. On physical examination at presentation, his general condition was good, he was conscious, his blood pressure was 135/75 mmHg, his body temperature was 36.9 °C, his pulse rate was 110/min, and his respiratory rate was 24/min. Two or more pitting edema and bifissure edema were detected in the pretibial region. Breath sounds were normal, and there were no abdominal ascites or scrotal edema. Cardiac examination revealed a pansystolic murmur to the apical 3/6 left axilla and a diastolic murmur in the left third intercostal space.

**Fig. 1:**
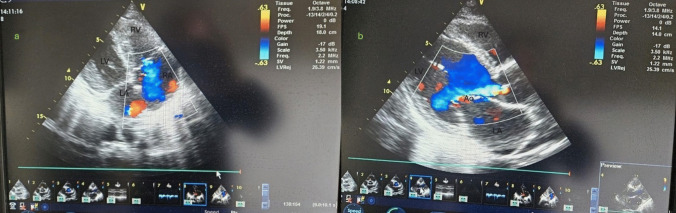
**a** Moderate tricuspid regurgitation. **b** Moderate eccentric aortic regurgitation. RV: Right ventricle; LV: Left ventricle; RA: Right atrium; LA: Left atrium; Ao: Aortic valve

Laboratory investigations revealed hemoglobin of 12.7 g/dL, leukocyte count of 8,440/mm^3^, platelet count of 251,000/mm^3^, erythrocyte sedimentation rate (ESR) of 42 mm/hour, C-reactive protein (CRP) of 1.9 mg/dL, and ASO level of 555 IU/mL. Kidney function tests showed BUN of 42 mg/dL, creatinine of 1.04 mg/dL, total protein of 5.7 g/dL, albumin of 3.6 g/dL, pro-BNP of 2216 pg/mL, and a decreased serum C3 level (0.14 g/L). Urinalysis revealed + 4 proteinuria, + 3 hematuria, and microscopic examination of the urine identified 468 dysmorphic erythrocytes per field. Urine, throat and blood cultures were obtained and all were negative. Anti-DNA, antinuclear antibodies, anti neutrophilic cytoplasmic antibodies (ANCA), double-stranded DNA antibody, anti-glomerular basement membrane and rheumatoid factor were negative. The patient was hospitalized with a preliminary diagnosis of nephritic syndrome, and furosemide therapy was initiated. Electrocardiography showed a PR interval of 0.20 s, and chest radiography revealed cardiomegaly and bilateral pleural effusion. Echocardiography revealed moderate aortic regurgitation, moderate tricuspid regurgitation, mild mitral regurgitation, and moderate to severe acute rheumatic carditis (Fig. 1). The patient was diagnosed with both APSGN and (ARF, and treatment was initiated with benzathine penicillin (1,200,000 U, IM) and prednisolone (1 mg/kg/day, oral) for a duration of eight weeks. By the third day of hospitalization, a decrease in pro-BNP levels was observed, and by the seventh day, the pro-BNP value had returned to the normal range. However, since the patient's arterial blood pressure remained elevated, antihypertensive therapy with amlodipine was initiated. On the 9th day of hospitalization, laboratory evaluation showed a rising trend in C3 levels. Concurrently, regression of pretibial edema was observed, leading to a reduction in the furosemide dose to 0.5 mg/kg. On the 15th day of hospitalization, the patient had no edema, and the serum C3 level was measured at 0.71 g/L. He was discharged with ongoing treatment including prednisolone, amlodipine, and furosemide.

At the first follow-up, regression of edema was observed, microscopic hematuria persisted, but proteinuria was not detected. The serum C3 level was found to have reached the normal range (1.03 g/L). Based on these findings, a gradual tapering of the prednisolone dose was planned. Echocardiography revealed significant improvement in cardiac findings, yet mild valvular regurgitation persisted. At the first month follow-up, no pathological findings were noted other than mild aortic regurgitation. Prednisolone treatment was terminated after a total of 8 weeks. The patient continued amlodipine and furosemide therapy along with benzathine penicillin prophylaxis administered every 21 days. At the three month follow-up, the patient's arterial blood pressure values remained stable, and amlodipine and furosemide treatments were discontinued. Microscopic hematuria and mild aortic regurgitation on echocardiography persisted.

**Fig. 2:**
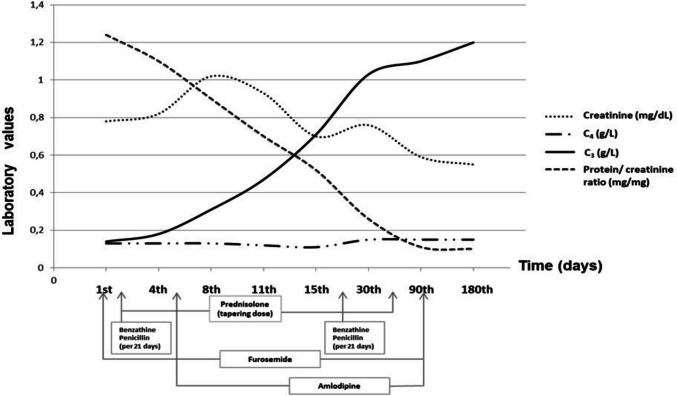
Laboratory changes and treatment protocols by day

At the six month follow-up, physical examination findings and all biochemical parameters, including complete urinalysis, were normal (Fig. 2), and mild aortic regurgitation persisted in echocardiography. Informed consent was obtained from the patient's parents.

## Methods and materials

### Study registration and search strategy

This article was registered in the International Prospective Register of Systematic Reviews (PROSPERO, registration number: CRD420251141001). We followed the reporting guideline from the Preferred Reporting Items for Systematic Reviews and Meta-Analyses [7].

Four electronic databases—PubMed, Scopus, Web of Science and Google Scholar—were systematically searched for articles published on or before September 15, 2025. The retrieval strategy for all databases is shown in Supplementary Table 1. The references listed in the included articles were also screened. Only studies published in English were included in our study.

### Eligibility criteria

Studies meeting the following criteria were included: (1) full-text articles available in English, (2) case reports, case series or letters to the editor, and (3) information provided on patient demographics, clinical presentation, laboratory findings, treatment and follow-up results. Articles that did not meet these criteria or were review articles without original case data were excluded.

### Study selection and data extraction

Two independent reviewers (TK and SY) conducted the literature search and removed duplicates. Two researchers independently screened the titles and abstracts to determine the potentially relevant studies that met the inclusion criteria. The initial screening was performed by evaluating titles and abstracts, while the remaining articles underwent full-text assessment based on the eligibility criteria. The same two researchers also independently assessed the full text, and a third researcher (AI) resolved any disagreements. Two reviewers (TK and SY) independently extracted data from eligible studies using a pre-specified form. The reference lists of all included studies, previous case reports and letters to the editor, were manually screened to identify additional studies.

### Quality assessment

All included studies were evaluated for risk of bias by two authors. For case series studies, we used the quality evaluation tool of case series study recommended by the National Institutes of Health [8]. Missing data management was handled as follows: descriptive analyses were performed using all available data; therefore, denominators vary across variables and are reported accordingly. No imputation was performed. For inferential analyses, complete-case analyses were conducted for each model/test, excluding participants with missing values in any variable required for that specific analysis.

### Outcome measurements

The main outcomes included age, clinical presentation, serum creatinine levels, proteinuria levels, serum complement component-3 (C3), kidney biopsy, follow-up duration, cardiac findings and treatments. We defined kidney recovery as the composite of (i) age-adjusted normal serum creatinine/eGFR; (ii) resolution of urinary abnormalities (no gross hematuria; microscopic hematuria resolved or markedly improved; proteinuria trace/negative); (iii) normalization of C3 by 8–12 weeks at follow-up.

## Results

**Fig. 3:**
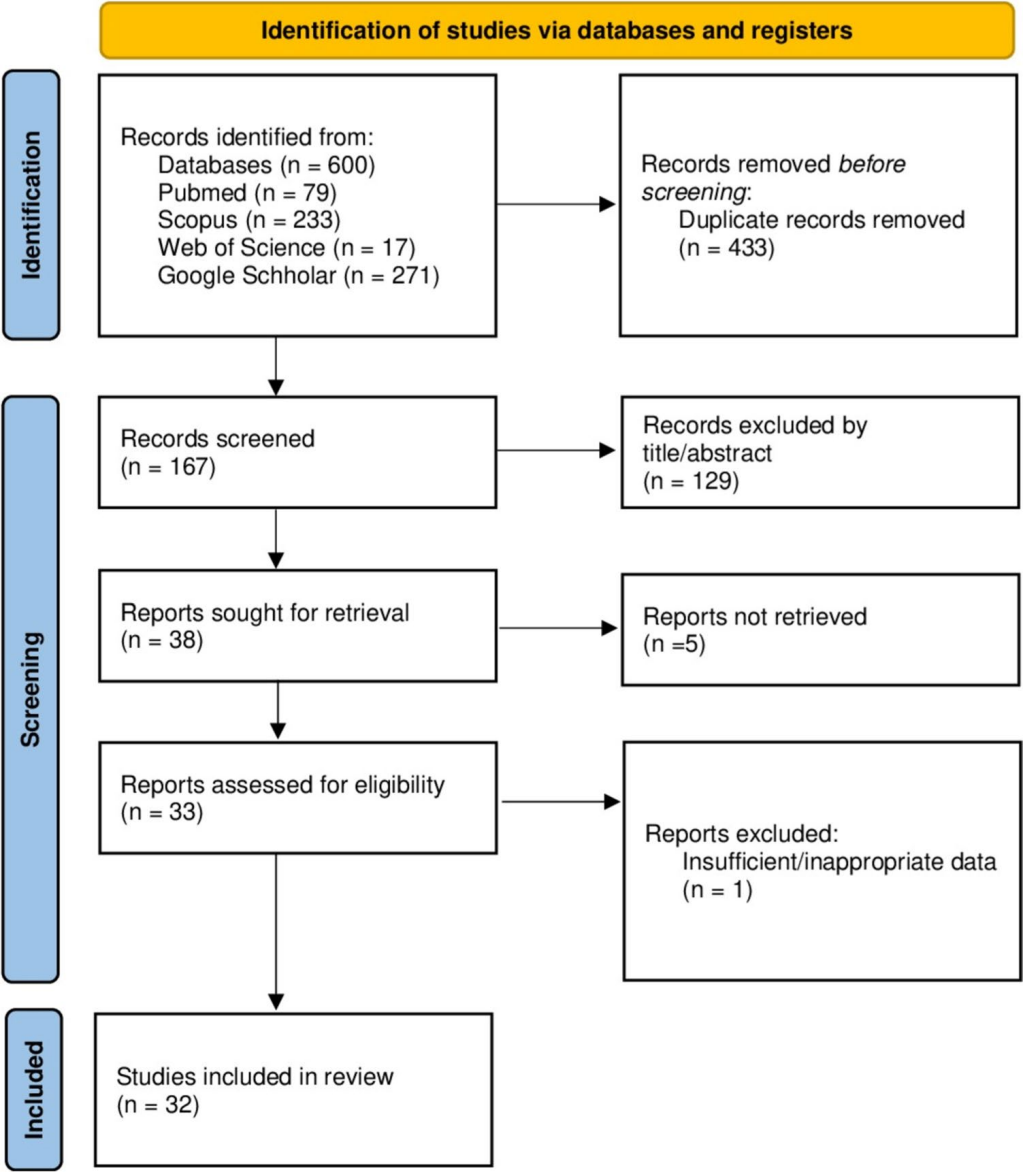
Study flow diagram

The initial search identified 600 articles. After removing duplicate articles, 167 abstracts and full texts were assessed against the inclusion criteria. Of these, 129 were excluded after screening their abstract. Finally, 32 studies were included in the systematic review [3–6, 9–34]. The study selection process is described in Fig. 3. Reasons for full-text exclusions are summarized in Supplementary Table 2.

Among the 36 cases examined, patient ages ranged from 3 to 47 years, with a median age of 10 years (Mean ± SD: 9.33 ± 3.79, IQR 10–14) and most of the patients were in the pediatric age group (31/36, 86.1%). The proportion of male patients was 50% (18/36) and the proportion of female patients was 50% (18/36). The most frequently reported clinical findings were edema (23/36, 63.8%), fever (20/36, 55.5%), hypertension (20/36, 55.5%), arthritis/arthralgia (17/36, 47.2%), hematuria (14/36, 38.8%), oliguria (4/36, 11.1%) and rash (4/36, 11.1%). Serum creatinine levels measured at presentation varied among cases, with a median of 1.3 mg/dL (Mean ± SD: 1.82 ± 1.28, IQR 0.9–2.2). Proteinuria levels ranged from + 1 to + 4, with the most common level among cases reporting values ​​being + 3 (12/29, 41.3%). C3 complement levels were found to be low in all reported cases (31/31, 100%). The median C3 level in cases with reported C3 levels was 0.17 g/L (Mean ± SD: 0.30 ± 0.22, IQR 0.11–0.51). The most common cardiac involvement was mitral regurgitation (MR, 32/36, 88.8%). In some cases, aortic regurgitation (AR, 14/36, 38.8%), tricuspid regurgitation (TR, 5/36, 13.8%), pericardial effusion (13.8%), left ventricular enlargement (8.3%) and pulmonary regurgitation (PR, 2/36, 5.5%) were also detected. Kidney biopsy was performed in a total of 16 cases (44.4%), and the pathological finding in all patients was reported as diffuse proliferative glomerulonephritis (16/16, 100%). In 5 patients, crescents accompanied diffuse proliferative GN (31.2%). IgA-dominant diffuse proliferative glomerulonephritis – the original authors classified it as APSGN based on low C3 and the clinical course; therefore, we included it in our analysis – was detected in one case. It was reported that antibiotic prophylaxis was initiated in 33 cases (91.6%) during treatment. Additionally, diuretics (28/36, 77.7%), aspirin (25/36, 69.4%), corticosteroids (prednisolone/methylprednisolone) (16/36, 44.4%) and ACE inhibitors (11/36, 30.5%) were administered in most cases. The median follow-up period of the cases was 11 months (Mean ± SD: 14.5 ± 16.88, IQR 6–12), and kidney function was completely recovered in all patients with a reported follow-up period except for one patient who continued to have microscopic hematuria (26/27, 96.2%). Cardiac sequelae persisted in 19/27 cases (70.3%), with mitral regurgitation being the most frequent finding (16/27, 59.2%). However, severe permanent sequela was rare, except for one patient who underwent mechanical valve replacements (Table 1). In terms of cumulative sequelae (kidney and/or cardiac), it was determined that age and gender did not reveal any difference.

**Table 1:** Clinical Characteristics of Patients with Concurrent APSGN and ARF

Case	Age/Gender	Clinical Presentation	Cardiac Findings	Baseline Creatinine (mg/dL)	Baseline Proteinuria Level	C3 Level (g/L) (C3 Status)	Renal Biopsy	Treatment	Follow-up Duration (Months)	Outcome (Renal/Cardiac)
Rus et al. (Slovenia) [35]	3 yrs, Male	Arthritis, fever, hematuria	Pericardial effusion, mild to moderate MR, mild AR	1.3	3 +	0.69	IgA dominant diffuse proliferative GN, crescents	Penicillin prophylaxis, steroid, dialysis, diuretics	48 months	Renal recovery; no cardiac sequelae
Chandrasekhara et al. (India) Case-1 [9]	3 yrs, Male	Hematuria, fever	MR	2.0	4 +	0.12	Diffuse proliferative GN	Penicillin prophylaxis, diuretics, aspirin	12 months	Renal recovery; mild MR persisted
Öner et al. (Turkiye) [10]	4 yrs, Male	Fever, Edema, HT	MR	3.1	2 +	0.43	Not performed	Penicillin prophylaxis, diuretics, aspirin	Not reported	Not reported
Fioretti et al. (Italy) [4]	4.5 yrs, Male	Fever, hematuria	Mild MR + AR	Normal range but no numerical data given	Not reported	0.17	Not performed	Penicillin prophylaxis, aspirin	Not reported	Not reported
Camcı et al. (Turkiye) [11]	4,5 yrs, Male	Arthritis, fever, edema, HT	Mild AR and moderate MR	0.49	Not Reported	0.09	Not performed	Diuretics, aspirin, digoxin, penicillin prophylaxis, ACE inhibitor	Not reported	Not reported
Vilija et al. (Lithuania) [12]	5 yrs, Female	Fever, edema, polyarthralgia	Severe MR, mild AR, pericardial effusion	Not reported	Not reported	0.79	Not performed	Penicillin prophylaxis, steroid, diuretics, ACE inhibitor	6 months	Renal recovery; no cardiac sequelae
Mattoo et al. (Saudi Arabia) [13]	6 yrs, Female	Edema, arthritis, rash, HT	Pericardial effusion, mild MR, Mild AR	2.2	Not Reported	0.53	Diffuse proliferative GN	Steroid, azathioprine, ACE inhibitor, penicillin prophylaxis	Not reported	Not reported
Çetin et al. (Turkiye) [14]	6.5 yrs, Male	Hematuria, HT	MR	0.8	2 +	0.20	Diffuse proliferative GN	Penicillin prophylaxis, diuretics, steroid, aspirin	6 months	Renal recovery; mild MR persisted
Arslansoyu Çamlar et al. (Turkiye) [15]	7 yrs, Female	Arthritis, fever, edema, HT	MR, mitral valve thickening, mild AR	0.71	4 +	0.45	Diffuse proliferative GN, crescents	Penicillin prophylaxis, steroid, ACE inhibitor, diuretics	10 months	Microscopic haematuria, no cardiac sequelae
Tabel et al. Case-1 (Turkiye) [16]	7 yrs, Female	Edema, oliguria, HT	Moderate MR and AR	1.8	3 +	0.08	Not performed	Penicillin prophylaxis, steroid, aspirin, diuretics, ACE inhibitor	12 months	Renal recovery; mild MR and AR persisted
Suresh & Chandran (India) [17]	8 yrs, Female	Edema, oliguria, arthritis, fever, hematuria, HT	Moderate MR, mild AR and TR	Not reported	1 +	low but no numerical data given	Not performed	Penicillin prophylaxis, aspirin, ACE inhibitor	2 months	Renal recovery; no cardiac sequelae
Kujala et al. (ABD) [18]	8 yrs, Female	Arthritis, fever, rash	Mild TR, left ventricule enlargement	2.6	2 +	0.72	Diffuse proliferative GN, crescents	Aspirin, penicillin prophylaxis, steroid	Not reported	Not reported
Chandrasekhara et al. (India) Case-2 [9]	9 yrs, Female	Edema	MR	1.2	3 +	0.10	Diffuse proliferative GN	Penicillin prophylaxis, diuretics, aspirin	6 months	Renal recovery; mild MR persisted
Said et al. (Saudi Arabia) [19]	9 yrs, Male	Rash, fever, edema	Not performed, only EKG and chest X-ray	2.2	3 +	0.27	Diffuse proliferative GN	Erythromycin (he has penicillin allergy), digoxin, diuretics, steroid	6 months	Renal recovery; no cardiac sequelae
Sumarno & Tjiang, (Indonesia) [20]	9 yrs, Female	Edema, oliguria, arthritis	Moderate MR, heart failure	1.0	1 +	Not reported	Not performed	Erythromycin, dopamine, diuretics, ACE inhibitor, aspirin	6 months	Renal recovery; mild MR persisted
Bisno et al. (USA) [3]	9 yrs, Male	Hematuria, edema	MR	1.0	3 +	0.10	Not performed	Penicillin prophylaxis, diuretics, aspirin	6 months	Renal recovery; mild MR persisted
Imanaka et al. (Japan) [21]	10 yrs, Male	Fever, polyarthralgia, edema	Mild MR	0.9	3 +	0.16	Diffuse proliferative GN, subepithelial hump	Penicillin prophylaxis, aspirin, steroid	12 months	Renal recovery; no cardiac sequelae
Tabel et al. Case-2 (Turkiye) [16]	10 yrs, Male	Cough, dyspnea, edema, HT	Significant MR	0.9	2 +	0.60	Not performed	Penicillin prophylaxis, aspirin, steroid, diuretics	6 months	Renal recovery; mild MR persisted
Lin et al. (Taiwan) [22]	10 yrs, Female	Edema, oliguria, fever, HT, rash	Mild MR and AR, tiny tricuspid, PR, pericardial effusion	Not reported	3 +	0,14	Diffuse proliferative GN	Aspirin, penicillin prophylaxis, diuretics, ACE inhibitor	6 months	Renal recovery; mild TR, mild PR
Matsell et al. (USA) [23]	10 yrs, Female	Hematuria	MR	1.0	2 +	0.18	Diffuse proliferative GN	Penicillin prophylaxis, diuretics, aspirin	6 months	Renal recovery; mild MR persisted
Chandrasekhara et al. Case-3 (India) [9]	10 yrs, Male	Hematuria, HT	MR	1.3	2 +	0.13	Not performed	Penicillin prophylaxis, diuretics, aspirin	12 months	Renal recovery; mild MR persisted
Bozabali et al. (Turkiye) [24]	11 yrs, Male	Arthritis, fever	Mild AR and MR	Not reported	Not reported	0.22	Not performed	Penicillin prophylaxis	Not reported	Not reported
Kobayashi et al. (Japan) [25]	11 yrs, Female	Edema, HT, fever	Mild MR and TR trivial AR and PR, pericardial effusion	0.8	4 +	0.10	Diffuse proliferative GN	Steroid, ACE inhibitor, penicillin prophylaxis	72 months	Renal recovery; no cardiac sequelae
Kula et al., case-1 (Turkiye) [6]	12 yrs, Male	HT	MR	0.7	3 +	0.22	Diffuse proliferative GN	Diuretics, aspirin, penicillin prophylaxis	12 months	Renal recovery; mild MR persisted
Adedoyin et al. (Nigeria) [26]	13 yrs, Female	Edema, HT	Severe MR	5.6	3 +	Not reported	Not performed	Diuretics, ACE inhibitor	12 months	Renal recovery; moderate MR persisted
Kula et al., case-2 (Turkiye) [6]	14 yrs, Female	Edema, HT, fever, arthralgia	MR, LV enlargement	3.4	2 +	0.44	Not performed	Penicillin prophylaxis, diuretics, aspirin	Not reported	Not reported
Vakiti et al. (India) [27]	14 yrs, Female	Edema, hematuria, HT	MR, TR	1.2	3 +	0.11	Not performed	Penicillin prophylaxis, diuretics, aspirin	12 months	Renal recovery; mild MR and TR persisted
Abdulkader et al. (India) [5]	15 yrs, Male	Fever, hematuria, arthritis	MR	1.7	2 +	0.45	Not performed	Penicillin prophylaxis, diuretics, aspirin	6 months	Renal recovery; mild MR persisted
Our case (Turkiye)	15 yrs, Male	Edema, HT, hematuria	Moderate to severe AR, moderate TR, mild MR	1.04	4 +	0.14	Not performed	Penicillin prophylaxis, diuretics, steroid	12 months	Renal recovery; significant cardiac improvement but mild AR persisted
Kwong et al. (USA) [28]	16 yrs, Female	Hematuria, arthritis	MR	0.6	1 +	0.23	Not performed	Penicillin prophylaxis, diuretics, aspirin	6 months	Renal recovery; mild MR persisted
Sieck et al. (USA) [29]	16 yrs, Male	Fever, arthritis	MR	1.4	2 +	0.20	Not performed	Penicillin prophylaxis, diuretics, aspirin	12 months	Renal recovery; mild MR persisted
Ben-Dov et al. case-1 (Israel) [30]	23 yrs, Female	Edema, hematuria, HT, arthritis, fever	Congestive heart failure, severe carditis	1.6	2 +	Not reported	Diffuse proliferative GN, crescents	Aspirin, diuretics, steroid, azathioprine, penicillin prophylaxis	60 months	Renal recovery; no cardiac sequelae
Nakauyaca et al. (Australia) [31]	29 yrs, Male	Fever, arthritis	Modarete MR, mild AR	4.51	Not reported	0.82	Not performed	Steroid, penicillin prophylaxis	24 months	Renal recovery; mechanical aortic and mitral valve replacements 7 months later
Akasheh et al. (Jordan) [32]	37 yrs, Male	Edema, HT, fever	Mild AR, enlarged left ventricular end diastolic diameter	3.48	3 +	0.36	Diffuse proliferative and exudative GN, crescent	Diuretics, digoxin, penicillin prophylaxis, aspirin, steroid, vasodilator	6 months	Not reported
Mikkelsen et al. (Denmark) [33]	44 yrs, Female	Edema, HT, hematuria	Modarete MR, PHT	4.3	Not reported	Not reported	Diffuse proliferative GN	Diuretics, beta blockers	Not reported	Not reported
Ghosh et al. (USA) [34]	47 yrs, Female	Edema, polyarthralgia	Severe MR	1.6	3 +	Not reported	Not performed	Diuretics, vasodilator, penicillin prophylaxis, steroid, aspirin	12 months	Renal recovery; moderate MR

*APSGN* Acute poststreptococcal glomerulonephritis, *ARF*: Acute rheumatic fever, *MR*: Mitral regurgitation, *AR*: Aortic regurgitation, *TR*: Tricuspid regurgitation, *PHT*: Pulmonary hypertension, *GN*: Glomerulonephritis, *HT*: Hypertension, *C3*: Complement component 3; yrs: years, *LV*: Left ventricule

## Discussion

The simultaneous occurrence of ARF and APSGN is a rare clinical challenge because both are distinct immune-mediated sequelae of streptococcal infection. In our synthesis of 36 published cases (including our own), most patients were pediatric (86.1%). Importantly, the data suggest a prognostic divergence between kidney and cardiac outcomes: kidney recovery was reported in 26/27 cases with follow-up (96.2%), whereas persistent cardiac sequelae were reported in 19/27 cases (70.3%). Mitral regurgitation was the most common residual lesion (16/27, 59.2%). Our patient followed the same pattern, with complete kidney recovery but persistent mild aortic regurgitation at the six month follow-up. This focused synthesis updates and extends the systematic review by Bertola et al. (2019), who identified 29 APSGN cases associated with extrarenal immune-mediated disorders, including APSGN/ARF co-occurrence [36]. By expanding the search window through 2025, we identified 36 cases and provide a more granular synthesis of long-term cardiac sequelae and therapeutic approaches.

The pathogenesis of this coexistence remains unclear. One hypothesis is that certain group A streptococcal strains may possess overlapping rheumatogenic and nephritogenic virulence determinants, suggesting that the classical dichotomy between “rheumatogenic” and “nephritogenic” strains may not be absolute. The fact that both diseases affect similar age groups—ARF typically between 5–15 years and APSGN between 4–14 years—may also contribute to their concurrent manifestation [37–39].

According to the case series by Wijaya et al., the annual incidence of PSGN in developing countries is approximately 9.5 per 100,000 population, whereas rheumatic heart disease (RHD) affected fewer than 5 million cases in 2013 [40]. These figures suggest that PSGN is more common than RHD. However, the frequency of cardiac involvement in PSGN cases is not entirely clear. The presence of cardiac findings in all cases in our study may reflect a select group of patients.

A central issue in managing these concurrent cases is the role of corticosteroids. The use of corticosteroids in APSGN is controversial; such therapy is generally reserved for unusual, severe presentations, including rapidly progressive glomerulonephritis (RPGN) evidenced by crescents on biopsy or refractory kidney failure [41]. However, in the context of coexisting ARF with significant carditis, steroids are often considered for their potent anti-inflammatory effects. In our literature review, corticosteroids were used in 44.4% of cases, often in patients with more severe presentations. In isolated ARF, anti-inflammatory therapy is a mainstay: high-dose aspirin or NSAIDs are typically used for mild to moderate carditis and arthritis. A systematic review assessed various therapies for carditis and their ability to prevent further cardiac damage; neither corticosteroids nor intravenous immunoglobulin (IVIG) were determined superior to aspirin therapy for this purpose [42]. Corticosteroids may help manage patients with severe acute RHD, marked by significant mitral regurgitation or persistent atrioventricular block [43]. Our patient, who presented with moderate-to-severe carditis, was treated with prednisolone, which was followed by significant clinical and echocardiographic improvement. Importantly, among the published concurrent APSGN/ARF cases, no irreversible worsening of kidney function or steroid-attributed uncontrollable hypertensive crises were reported; however, this observation should be interpreted cautiously given the retrospective nature of case reports and the possibility of under-reporting of adverse events. When steroids are used for severe carditis in the setting of nephritic syndrome, meticulous monitoring of blood pressure and fluid balance remains essential. Nevertheless, due to the limited number of cases, it is difficult to conclusively determine whether steroid therapy significantly alters long-term outcomes.

The persistence of cardiac sequelae appears to be multifactorial. Factors such as advanced age, delays in diagnosis and treatment, and the initial severity of cardiac involvement can negatively impact recovery. For instance, a case with initial severe mitral regurgitation showed persistence of moderate regurgitation during follow-up [26]. Furthermore, genetic predisposition and autoimmune mechanisms play a significant role in the development of RHD [44]. Long-term antibiotic prophylaxis is critical for preventing recurrent attacks and further cardiac damage, although adherence and duration were not uniformly reported in the reviewed cases.

In the literature, the association of PSGN and cardiac involvement is mostly discussed in the context of ARF. As noted in the study by Maness et al. (2018), carditis, which is one of the major Jones criteria for ARF, is observed in approximately 50–60% of cases [45]. The same study compared ARF with post-streptococcal reactive arthritis and emphasized that cardiac involvement is more pronounced in ARF. The majority of the cardiac findings in the cases we examined appear to be compatible with the diagnostic criteria for ARF. This suggests that cardiac symptoms observed concurrently with PSGN may often be due to ARF. Therefore, cardiac findings associated with PSGN should not be considered an innocuous condition, and patients should be carefully monitored for carditis. A new heart murmur (especially mitral or aortic regurgitation), tachycardia out of proportion to fever, cardiomegaly on imaging, or a prolonged PR interval on ECG in a child with nephritic syndrome are red flags pointing to rheumatic carditis concurrent with APSGN. In addition, hypocomplementemia (low C3)—a characteristic feature of APSGN—was reported in all cases with available complement data in our dataset (31/31); thus, a low C3 level may help support the diagnosis of APSGN in patients whose edema and cardiomegaly could otherwise be attributed solely to cardiorenal volume overload. The risk of sequelae depends not only on the nature of the disease but also on various factors, including the patient's age, timing of treatment initiation, initial disease severity, and genetic predisposition. Prospective, large-sample studies are needed to better understand this association and optimize clinical management.

From a prognostic perspective, although ARF can generally be treated during the acute phase, it carries a risk of permanent cardiac valvular damage due to the development of carditis in approximately 50–60% of cases [45]. Patients who had rheumatic carditis require regular cardiology evaluations (clinical and echocardiographic) well beyond 1–2 years, as valvular damage may persist or progress. Guidelines for ARF advise prolonged antibiotic prophylaxis to prevent recurrence: for example, patients with residual rheumatic valvular disease should receive penicillin prophylaxis for at least 10 years or until about age 40, whichever is longer [46].

In contrast, the short-term prognosis of APSGN in children is generally favorable; most cases achieve full recovery or significant improvement, and progression to kidney failure has been reported in only about 2% of cases [47]. Nevertheless, follow-up for at least 6–12 months is recommended to ensure normalization of blood pressure, resolution of hematuria/proteinuria, and return of complement levels to normal [48]. Patients with severe initial glomerular injury or those in older age groups (e.g. adolescents or adults with PSGN) should be monitored beyond 2 years for late hypertension, persistent urinary abnormalities, or declining kidney function [48]. This suggests that ARF may cause cardiac sequelae and APSGN may cause renal sequelae, but permanent kidney damage is less common. Any patient with rheumatic cardiac involvement will require long-term (often multiyear to lifelong) cardiologic follow-up and prophylaxis, and those with severe or atypical APSGN should receive extended nephrologic follow-up beyond the usual 6–24 months to ensure no chronic kidney disease ensues.

## Limitations

This study has several limitations. First, as a retrospective review of case reports, it is subject to publication bias, where more severe or unusual cases are more likely to be reported. Second, the total number of cases is small, which limits the statistical power of our conclusions. In our systematic review, we were unable to access the full text of 5 of the 38 articles (13.1%). These unretrieved reports were published between 1981 and 1998; therefore, their exclusion may have limited the completeness of historical data capture, and we consider this a limitation. Finally, there was variability in the reporting of treatment protocols and the duration of follow-up across the cases, making direct comparisons challenging. Additionally, the PROSPERO registration was completed retroactively, as the work was initially conceived as a case-based review; this may be considered a limitation.

## Conclusion

The coexistence of ARF and APSGN represents a rare but clinically important entity requiring multidisciplinary management. In most reported cases, kidney recovery is favorable; however, persistent valvular disease is frequent and is likely the main driver of long-term morbidity. Accordingly, clinicians should maintain heightened suspicion for rheumatic carditis in patients presenting with APSGN and systemic symptoms (e.g., fever, arthralgia/arthritis) or cardiac findings, and should consider echocardiography and ECG evaluation even when clinical signs are subtle. Management should balance control of nephritic fluid overload with anti-inflammatory therapy when severe carditis is present, and long-term follow-up should prioritize cardiology surveillance and guideline-based secondary prophylaxis. Future prospective studies are needed to better delineate pathogenesis and optimize treatment strategies.

## Supplementary Information

Below is the link to the electronic supplementary material.

ESM 1 (DOCX 15.0 KB)

ESM 2 (DOCX 17.1 KB)

Graphical abstract (PPTX 79.9 KB)

## Data Availability

All data relevant to this case report and literature review are included in the article. No additional datasets were generated or analyzed.
