# Supplementary material for: Coexistence of acute post-streptococcal glomerulonephritis and acute rheumatic fever: case report and systematic review
Source: Pediatr Nephrol. 2026 Feb 17;41(9):2863–75. doi: 10.1007/s00467-026-07189-7 (PMC13424215; doi:10.1007/s00467-026-07189-7)
Supplement: Supplementary file 1 — (DOCX 15.0 KB) [file 467_2026_7189_MOESM1_ESM.docx]

| **Supp Table 1.** Number of records identified through searching electronic databases |
| --- |
| **PubMed** (15/09/2025) |
| ((((((Acute Poststreptococcal Glomerulonephritis) OR (Poststreptococcal Nephritis)) OR (Poststreptococcal Glomerulonephritis)) OR (Post-streptococcal Nephritis)) OR (acute post-streptococcal glomerulonephritis)) AND ((((Acute Rheumatic Carditis) OR (Rheumatic Heart Disease)) OR (Acute Rheumatic Fever)) OR (Rheumatic Fever))) AND ((((Concurrent) OR (Occurrence)) OR (Concomitant)) OR (Coexistence)) |
| **Results**: 79 |
| **Scopus** (15/09/2025) |
| ALL ( "Acute Poststreptococcal Glomerulonephritis" OR "Poststreptococcal Nephritis" OR "Poststreptococcal Glomerulonephritis" OR "Post-streptococcal Nephritis" OR "acute post-streptococcal glomerulonephritis" AND "Acute Rheumatic Carditis" OR "Rheumatic Heart Disease" OR "Acute Rheumatic Fever" OR "Rheumatic Fever" OR "acute rheumatic carditis" AND "Concurrent " OR "Occurrence" OR "Concomitant" OR "Coexistence") |
| **Results:** 233 |
| **Web of Science** (15/09/2025) |
| Query #5  ALL=("Acute Poststreptococcal Glomerulonephritis" OR "Poststreptococcal Nephritis" OR "Poststreptococcal Glomerulonephritis" OR "Post-streptococcal Nephritis" OR "acute post-streptococcal glomerulonephritis")  Query #6  ALL=("Acute Rheumatic Carditis" OR "Rheumatic Heart Disease" OR "Acute Rheumatic Fever" OR "Rheumatic Fever" OR "acute rheumatic carditis")  Query #7  ALL=("Concurrent " OR "Occurrence" OR "Concomitant" OR "Coexistence")  Last search version: #5 AND #6 AND #7  (<https://www.webofscience.com/wos/woscc/summary/5e8d657d-1271-47cd-84a6-90af7267e806-018f708bd1/relevance/1>) |
| **Results:** 19 |
| **Google Scholar** (15/09/2025) |
| coexistence "Acute Poststreptococcal Glomerulonephritis" OR "Poststreptococcal Nephritis" OR "Poststreptococcal Glomerulonephritis" OR "Post-streptococcal Nephritis" OR "acute post-streptococcal glomerulonephritis" AND "Acute Rheumatic Carditis" OR "Rheumatic Heart Disease" OR "Acute Rheumatic Fever" OR "Rheumatic Fever" OR "acute rheumatic carditis" |
| **Results:** 271 |
