# Supplementary material for: Coexistence of acute post-streptococcal glomerulonephritis and acute rheumatic fever: case report and systematic review
Source: Pediatr Nephrol. 2026 Feb 17;41(9):2863–75. doi: 10.1007/s00467-026-07189-7 (PMC13424215; doi:10.1007/s00467-026-07189-7)
Supplement: Supplementary file 2 — (DOCX 17.1 KB) [file 467_2026_7189_MOESM2_ESM.docx]

**Supp Table 2. Excluded studies**

| **Study** | **Reason for exclusion** |
| --- | --- |
| Gibney R, Reineck HJ, Bannayan GA, Stein JH (1981) Renal lesions in acute rheumatic fever. Ann InternMed 94:322–326. https://  doi.org/10.7326/0003-4819-94-3-322 | No full text |
| Kakkera DR, Khan AJ, Bastawros MN, Lao J, Nudel DB (1998) Acute rheumatic pancarditis associated with poststreptococcal acute glomerulonephritis: a patient report. Clin Pediatr (Phila) 37:569–572. https://doi.org/10.1177/000992289803700909 | No full text |
| Castillejos G, Padilla L, Lerma A, González S, Reyes PA (1985) Coincidence of acute rheumatic fever and acute post streptococcal glomerulonephritis. J Rheumatol 12:587–589 | No full text |
| Narula AS, Mishra A, Anand AC, Gupta HS, SinghW(1992) Post streptococcal glomerulonephritis co-existing with acute rheumatic fever - a case report. J Assoc Physicians India 40:685–686 | No full text |
| Ben-Dov I, Berry EM, Kopolovic J (1985) Poststreptococcal neprhitis and acute rheumatic fever in two adults. Arch Intern Med 145:338–339. <https://doi.org/10.1001/archinte.1985>. 00360020182029 | No full text for case-2 |
| Sinha R, Al-AlSheikh K, Prendiville J, Magil A, Matsell D. Quiz page. Acute rheumatic fever with concomitant poststreptococcal glomerulonephritis. Am J Kidney Dis. 2007 Jul;50(1):A33-5. doi: 10.1053/j.ajkd.2007.04.001. PMID: 17687804. | Insufficient/inappropriate data/Wrong study type |
