# Supplementary material for: Coexistence of acute post-streptococcal glomerulonephritis and acute rheumatic fever: case report and systematic review
Source: Pediatr Nephrol. 2026 Feb 17;41(9):2863–75. doi: 10.1007/s00467-026-07189-7 (PMC13424215; doi:10.1007/s00467-026-07189-7)
Supplement: Supplementary file 3 — (PPTX 79.9 KB) [file 467_2026_7189_MOESM3_ESM.pptx]

## Slide 1
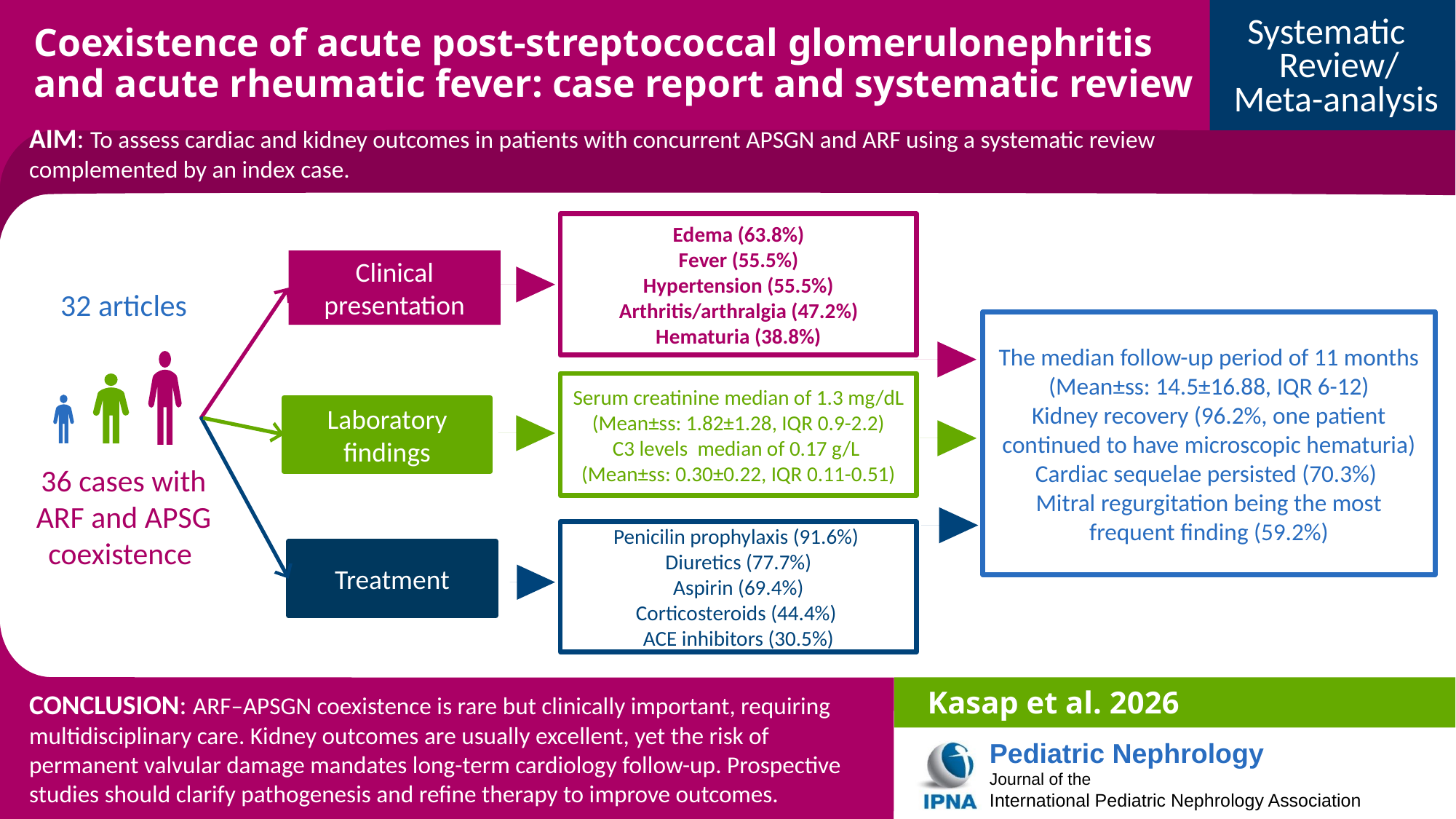

Coexistence of acute post-streptococcal glomerulonephritis and acute rheumatic fever: case report and systematic review
AIM: To assess cardiac and kidney outcomes in patients with concurrent APSGN and ARF using a systematic review complemented by an index case.
Edema (63.8%)
Fever (55.5%)
Hypertension (55.5%)
Arthritis/arthralgia (47.2%)
Hematuria (38.8%)
Clinical presentation
32 articles
The median follow-up period of 11 months (Mean±ss: 14.5±16.88, IQR 6-12)
Kidney recovery (96.2%, one patient continued to have microscopic hematuria)
Cardiac sequelae persisted (70.3%)
Mitral regurgitation being the most frequent finding (59.2%)
Serum creatinine median of 1.3 mg/dL
(Mean±ss: 1.82±1.28, IQR 0.9-2.2)
C3 levels median of 0.17 g/L
(Mean±ss: 0.30±0.22, IQR 0.11-0.51)
Laboratory findings
36 cases with ARF and APSG coexistence
Penicilin prophylaxis (91.6%)
Diuretics (77.7%)
Aspirin (69.4%)
Corticosteroids (44.4%)
ACE inhibitors (30.5%)
Treatment
Kasap et al. 2026
CONCLUSION: ARF–APSGN coexistence is rare but clinically important, requiring multidisciplinary care. Kidney outcomes are usually excellent, yet the risk of permanent valvular damage mandates long-term cardiology follow-up. Prospective studies should clarify pathogenesis and refine therapy to improve outcomes.
